# Visualisation and Identification of the Interaction between STIM1s in Resting Cells

**DOI:** 10.1371/journal.pone.0033377

**Published:** 2012-03-16

**Authors:** Jun He, Tao Yu, Jingying Pan, He Li

**Affiliations:** 1 Division of Histology and Embryology, Tongji Medical College, Huazhong University of Science and Technology, Wuhan, China; 2 Clinic Laboratory of Wuhan Children’s Hospital, Wuhan, China; Russian Academy of Sciences, Institute for Biological Instrumentation, Russian Federation

## Abstract

Store-operated Ca^2+^ channels are a major Ca^2+^ entry pathway in nonexcitable cells, which drive various essential cellular functions. Recently, STIM1 and Orai proteins have been identified as the major molecular components of the Ca^2+^ release-activated Ca^2+^ (CRAC) channel. As the key subunit of the CRAC channel, STIM1 is the ER Ca^2+^ sensor and is essential for the recruitment and activation of Orai1. However, the mechanisms in transmission of information of STIM1 to Orai1 still need further investigation. Bimolecular fluorescence complementation (BiFC) is one of the most advanced and powerful tools for studying and visualising protein-protein interactions in living cells. We utilised BiFC and acceptor photobleaching fluorescence resonance energy transfer (FRET) experiments to visualise and determine the state of STIM1 in the living cells in resting state. Our results demonstrate that STIM1 exists in an oligomeric form in resting cells and that rather than the SAM motif, it is the C-terminus (residues 233–474) of STIM1 that is the key domain for the interaction between STIM1s. The STIM1 oligomers (BiFC-STIM1) and wild-type STIM1 colocalised and had a fibrillar distribution in resting conditions. Depletion of ER Ca^2+^ stores induced BiFC-STIM1 distribution to become punctate, an effect that could be prevented or reversed by 2-APB. After depletion of the Ca^2+^ stores, BiFC-STIM1 has the ability to form puncta that colocalise with wild-type STIM1 or Orai1 near the plasma membrane. Our data also indicate that the function of BiFC-STIM1 was not altered compared with that of wild-type STIM1.

## Introduction

Protein–protein interactions play a pivotal role in the cellular signalling network. Monitoring protein-protein interactions in living cells is extremely useful for elucidating the dynamics and mechanisms of biological processes. In the past few decades, numerous methods [1], [2] have been developed to investigate protein–protein interactions. Among these methods, fluorescence resonance energy transfer (FRET) and protein fragment complementation are available for studying protein interactions in living cells. Specifically, the recently developed bimolecular fluorescence complementation (BiFC) assay is widely used because of its simplicity and high sensitivity [3]. The principle of BiFC is to split a fluorescent protein (FP) into two non-fluorescent fragments and fuse each fragment to one of a pair of interacting (test) proteins. Upon interaction of the two proteins, the two non-fluorescent fragments are brought into close proximity so that an intact FP is reconstituted [4], and fluorescence can be detected.

In electrically non-excitable cells, Ca^2+^ influx is mainly via store-operated Ca^2+^ channels (SOCs) that play important roles in the control of gene expression, cell growth and differentiation, secretion, Ca^2+^ homeostasis, and apoptosis [5], [6]. The best-characterised store-operated current, which was initially discovered in mast cells [7] and has since been recorded in several cell types [5], [6], [8], is the Ca^2+^ release-activated Ca^2+^ current (I_CRAC_). As the key subunit of CRAC channels, ER-resident protein STIM1 controls the opening of these channels. STIM1 is distributed throughout the ER; depletion of Ca^2+^ stores triggers the redistribution of STIM1 to sites of close apposition between the ER and the plasma membrane. This redistribution recruits Orai1 to STIM1 puncta, whereby the interaction of STIM1 and Orai1 can lead the opening of CRAC channels for Ca^2+^ influx to ER. Therefore, STIM1 is an ER Ca^2+^ sensor and is essential for the recruitment of Orai1 to puncta and activation of SOCs[6], [8], [9]–[12]. However, the cellular machinery modulating Orai1-STIM1 interactions remains poorly understood. Recently, Srikanth et al. identified a novel Ca^2+^ binding protein, CRACR2A that could regulate store-operated Ca^2+^ entry (SOCE) [13], [14]. The study shows that CRACR2A is an enhancer of SOCE, which stabilizes the interaction between Orai and STIM in the absence of Ca^2+^, thereby enhancing CRAC channel activity. As intracellular Ca^2+^ levels rise, CRACR2A binds Ca^2+^ and triggers SOCE inactivation by dissociating from the Orai–STIM complex [13], [14]. The discovery of CRACR2A provides a new insight into the regulation of STIM and Orai.

Previous studies suggest that STIM1 dimerises or oligomerises in the absence of Ca^2+^ and that it exists only in a monomeric form in the resting state in vitro [15], [16]. However, other reports indicate that STIM1 does not exist in a monomeric form under Ca^2+^-replete conditions (resting cells) because both endogenous and overexpressed STIM1–STIM1 and STIM1–STIM2 complexes can be readily co-immunoprecipitated from store-replete cell lysates[10], [17]–[19]. All of the above results were obtained from in vitro experiments. However, the state of STIM1 in living cells in resting conditions remains unexplored, which is important for better understanding how STIM1 works. STIM1 comprises a transmembrane domain that resides in the membrane of the ER, an N terminus that resides in the ER lumen, and a cytosolic C terminus. The N- terminus of STIM1 consists of an EF-hand and a sterile α motif (SAM) and is responsible for Ca^2+^ sensing. The C-terminus of STIM1 contains two coiled-coil regions that overlap with an ezrin-radixin-moesin (ERM)-like domain, followed by a serine/proline and a lysine-rich region. The functions of the C- and N- terminal domains of STIM1 need further investigation.

In the current study, BiFC was utilised for the first time to identify and visualise STIM1 in the living cells in resting state. STIM1 was found in an oligomeric form in cells in a resting state. The STIM1 oligomer (BiFC-STIM1) was identical to wild-type STIM1 in its morphological characteristics, dynamics, response to drugs and function. We have also confirmed that the key domain for the interaction between STIM1s in resting cells is not the SAM motif but the C-terminus (residues 233–474).

## Materials and Methods

### Cell culture and transfection

HEK293T(ATCC) cells were cultured in Dulbecco’s Modified Eagle’s Medium (DMEM) containing 10% heat inactivated foetal bovine serum, 50 U/ml penicillin, and 50 mg/ml streptomycin. HEK293T cells were maintained at 37°C in a humidified incubator set at 5% CO_2_. Cells were transfected with Lipofectamine 2000 (Invitrogen) as per the manufacturer’s instructions. Briefly, HEK293T cells were plated onto 30-mm round glass coverslips in a 6-well plate. The following day, cells were transfected with various plasmids according to the experimental protocol. Six hours later, the medium bathing the cells was replaced with complete DMEM, and the cells were maintained in culture overnight. After 24 h, the experiments were carried out on the transfected cells plated onto 30 mm round glass coverslips mounted in a Teflon chamber.

### Plasmid construction

mCherry-STIM1 was obtained from the laboratory of Richard S. Lewis, Stanford University, and the pHluorin-STIM1 from Pingyong Xu, Chinese Academy of Sciences. Full-length human Orai1 was amplified by PCR from a human placenta cDNA library (Invitrogen) and subcloned into pEYFP-N1 or pECFP-N1 plasmid. BiFC vectors VN173-ST1, VC155-ST1, VN173-ΔSAM, VC155-ΔSAM, VN173-ΔC, VC155-ΔC (where ST1 represents full length STIM1, ΔSAM indicates the SAM domain that was deleted and ΔC indicates that residues 233–474 were deleted) were constructed by the method previously described [4]. In brief, to express these proteins in mammalian cells, the sequences encoding STIM1 were fused to sequences encoding the FP Venus residues 1–173 (VN) or residues 155–238 (VC). All of the constructs were verified by sequencing.

### Solutions and chemicals

In confocal imaging experiments, we used standard extracellular Ringer’s solution containing the following (in mM): 150 NaCl, 5 KCl, 1.8 CaCl_2_, 1 MgCl_2_, 8 glucose, and 10 Hepes (pH 7.4, adjusted with NaOH). For the Ca^2+^-free Ringer’s solution, CaCl_2_ was replaced with 1 mM EGTA and 2 mM MgCl_2_. Stock solutions of thapsigargin (TG) and 2-APB were prepared in Me_2_SO at a concentration of 1 mM. Fluo-4-AM was purchased from Invitrogen. Unless otherwise specified, all of the reagents and chemicals were from Sigma-Aldrich.

### Western immunoblots

HEK293T cells was homogenized in NP40 buffer (50 mM Tris pH 7.4. 50 mM NaCl, 0.1% Triton X-100, 1% NP40, and protease inhibitor cocktail Pierce 78430 and 1 mM PMSF, Sigma P-7626). Cell lysates from cultures were sonicated and protein concentrations were determined. An equal amount of protein (30 µg/30 µl/lane) was separated on SDS-PAGE 10% gels. Proteins transferred to nitrocellulose were blocked in 5% non-fat dry milk in PBS for 30 min then incubated in primary antibodies in 3% BSA/PBS overnight at 4^○^C. Following incubation, blots were washed and secondary HRP-conjugated antibodies (Jackson Immuno-Resaerch) were added in 5% milk for 1 h. Blots were visualized using SuperSignal ECL (Pierce).

### Confocal microscopy imaging and Photobleaching

The experiments were performed using an Olympus FV500 laser scanning confocal microscopy system (Olympus, Tokyo). Coverslips containing cells were placed in a perfusion chamber on the stage of an inverted Olympus I×70 microscope. Data acquisition was performed in the sequential line mode for the best spatiotemporal reliability. The sample area was scanned at resolution of 512×512 pixels. The eCFP and eYFP were excited at 458 and 514 nm, respectively. Photobleaching experiments were performed using the same microscope with a 10 mW, 514 nm line of an argon laser. A 15 s pulse was used to locally photobleach ≈98% of the YFP intensity. All of the experiments were performed at 22°C–25°C.

### Acceptor-photobleaching-based FRET measurement

To determine FRET efficiencies, we used the acceptor photobleaching method. In the photobleaching experiments, a 15 s pulse of high-intensity laser at 514 nm was applied to bleach the YFP signal acceptor in a whole cell. A series of pre-bleaching and post-bleaching CFP (donor) fluorescence intensities were recorded. If any interactions leading to energy transfer were present in the cell, photobleaching of the acceptor would lead to an increase in donor fluorescence because it would no longer be quenched by the acceptor. The energy transfer efficiency (E) was calculated from the equation: 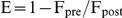



Here, F_pre_ and F_post_ stand for the fluorescence intensity of the donor before and after acceptor photobleaching, respectively.

### Intracellular Ca^2+^ Measurements

Cells were loaded with 2 µM fura-2/AM (Invitrogen) at 37°C for 30 min in standard Ringer’s solution. [Ca^2+^]i was measured using a dual-wavelength excitation (340/380 nm) photometry system on an inverted microscope (TE2000, Nikon, Japan) equipped with a polychromatic xenon light source (TILL photonics, Germany). The emission was collected at 510 nm with a photodiode controlled by the TILL photometry system and X-Chart extension of Pulse software (HEKA, Lambrecht, Germany). Ca^2+^ fluctuations are reported as the ratio of fluorescence emission at the two excitation wavelengths. Cells transfected alone with eYFP-STIM1 or cotransfected with VN173-ST1 and VC155-ST1 were chosen based on their fluorescence when excited at 514 nm.

## Results

### Identification of the oligomers state of STIM1 in resting cells by BiFC

**Figure 1:**
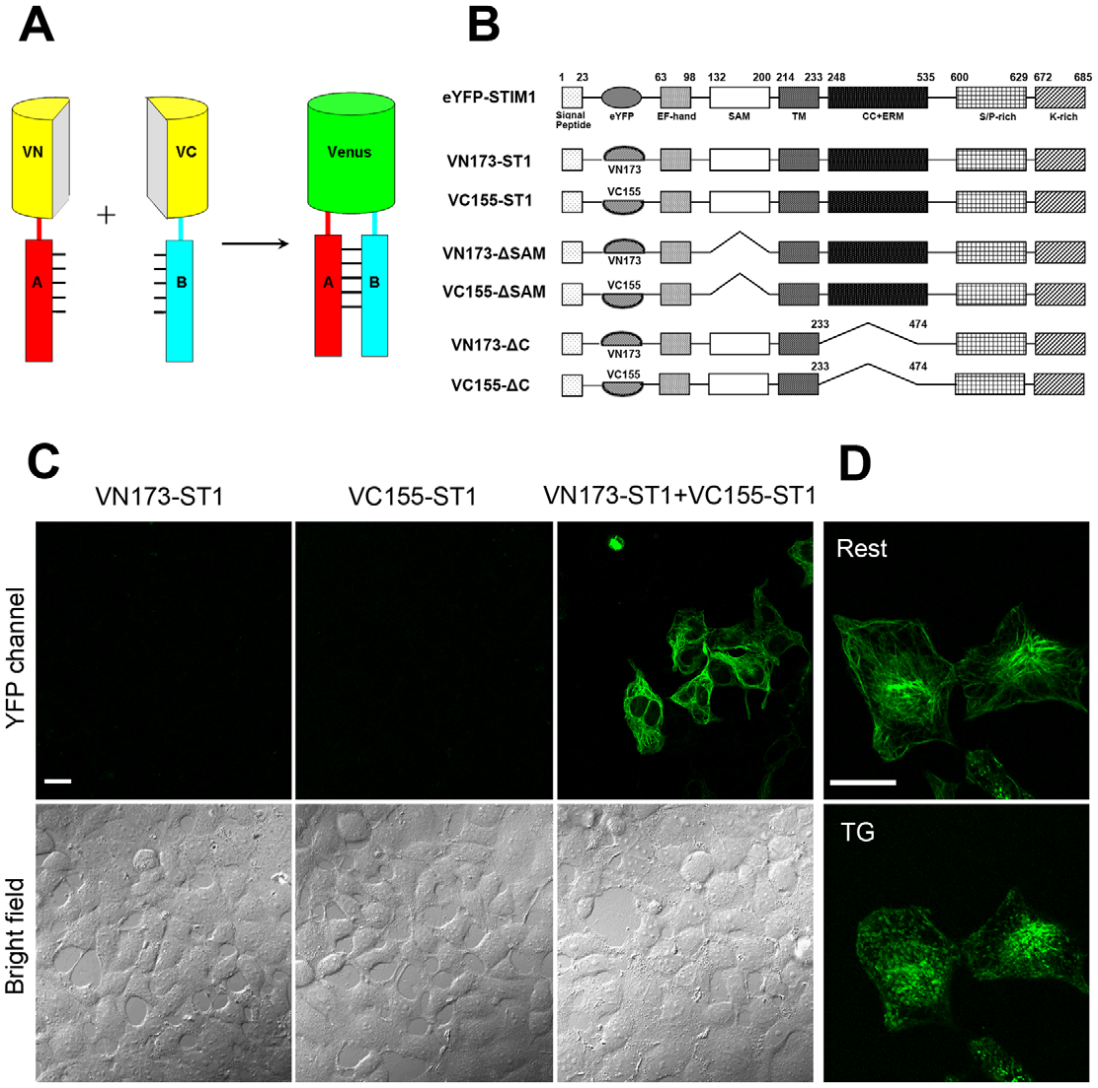
Visualisation of the STIM1-STIM1 interaction using a Venus-based BiFC system in resting mammalian cells. Part A shows the principles of the Venus BiFC system. The two non-fluorescent fragments of Venus, VN (N terminus of Venus) and VC (C terminus of Venus), are each fused to one of a pair of interacting (test) proteins, A and B. The fusion proteins, VN-A and VC-B, do not fluoresce when expressed separately. If proteins A and B interact or associate, the two fluorescent fragments are brought together, and this facilitates reconstruction of the fluorescent protein. Part B is a schematic view of the fusion protein constructs used in this study. WT-STIM1 and STIM1 mutants were fused to the N- and C-terminal fragments of Venus. The functional domains of STIM1 include an EF-hand, a SAM domain, a transmembrane domain, a coiled-coil (CC) region, an ERM domain, a Ser/Pro-rich domain (SP), and a polylysine residue region (K). Part C shows images of HEK293T cells 24 h after transfection with plasmids encoding VN173-ST1, VC155-ST1 or VN173-ST1/VC155-ST1 (full length STIM1). VN173-ST1 or VC155-ST1 expressed alone did not emit fluorescence under excitation, but coexpression of VN173-ST1 and VC155-ST1 produced strong yellow fluorescence emission. Part D shows HEK293T cells coexpressing VN173-ST1 and VC155-ST1 under resting and TG-stimulated conditions. Coexpression of VN173-ST1 and VC155-ST1 produced fluorescence that displayed a fibrillar distribution in resting cells and that became punctate and localised to the ER-PM junctions following stimulation of cells with 2 uM TG. Scale bars, 20 µm.

A BiFC system based on the FP Venus was used to detect STIM1-STIM1 interactions in living cells (Fig. 1). VN173 and VC155 were brought into close proximity (allowing them to reconstitute an intact Venus molecule) when two STIM1 molecules dimerize, making it possible to detect the formation of STIM1 homodimers by fluorescence readout. As shown in Fig. 1C, expression of VN173-ST1 or VC155-ST1 alone did not produce any fluorescence emission. However, coexpression of VN173-ST1 or VC155-ST1 in HEK293T cells produced strong yellow fluorescence, indicating the presence of STIM1 oligomers. The fluorescence exhibited a fibrillar distribution in resting cells. We named the VN173-ST1 and VC155-ST1 oligomers BiFC-STIM1. BiFC-STIM1 localised predominantly to the ER, similar to the distribution of wild-type GFP-STIM1. Ca^2+^ store depletion caused the STIM1 oligomers (BiFC-STIM1) to relocate to ER-PM junctions, where they were visible as puncta (Fig.1D); this is consistent with the punctate distribution of wild-type GFP-STIM1 upon Ca^2+^ store depletion.

### The C-terminal of STIM1 (233–474) plays a major role in oligomerisation of STIM1 in resting cells

**Figure 2:**
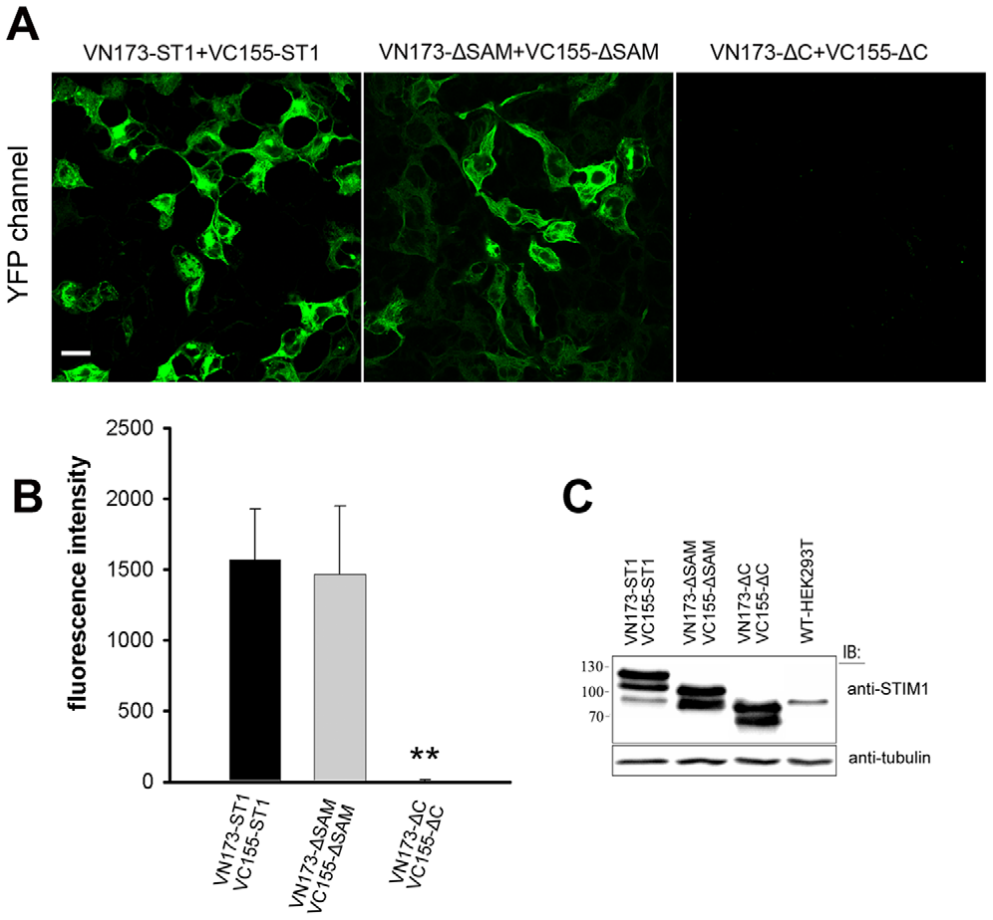
A C-terminal region of STIM1 (233–474) is critical for the oligomerisation of STIM1 in resting cells. Part A shows images of HEK293T cells cotransfected with the VN173-ST1 and VC155-ST1, VN173-ΔSAM and VC155-ΔSAM, or VN173-ΔC and VC155-ΔC constructs. Images were acquired 24 h after transfection. Part B shows quantitative analysis of Venus-based BiFC efficiency measured from experiments such as those shown in Part A. Part C shows that VN173-ST1 and VC155-ST1, VN173-ΔSAM and VC155-ΔSAM, or VN173-ΔC and VC155-ΔC constructs were cotransfected into HEK293T cells, respectively, and these proteins were detected by immunoblotting with anti-STIM1 Ab(Upper). Tubulin protein was detected as a loading control (Lower). All data are given as mean±S.D. (n>50). The statistical significance was evaluated using a two-tailed Student’s t-test when compared with the combination of VN173-ST1 and VC155-ST1. ST1, ΔC and ΔSAM represent full length STIM1, truncated STIM1-ΔC (Δ233–474) and STIM1-ΔSAM (Δ132–200), respectively. The significance level indicated is as follows: **P<0.001. Scale bar, 20 µm.

To identify the domain required to support the STIM1-STIM1 interaction in resting cells, we constructed two pairs of mutant STIM1 expression vectors, VN173-ΔSAM/VC155-ΔSAM and VN173-ΔC/VC155-ΔC (Δ233–474) (Fig. 1B). As shown in Fig. 2A, coexpressed VN173-ΔSAM and VC155-ΔSAM fragments exhibited yellow fluorescence that showed a similar intensity to that of coexpressed VN173-ST1/VC155-ST1 (p>0.05). However, cells cotransfected with VN173-ΔC and VC155-ΔC did not exhibit yellow fluorescence (Fig. 2A, B). These results show that STIM1 exists as oligomer in resting cells and that the key domain for STIM1 oligomerisation is not the SAM motif but the residues 233–474 within the C-terminal of STIM1.

### Detection of STIM1 interactions in living cells by FRET experiment

**Figure 3:**
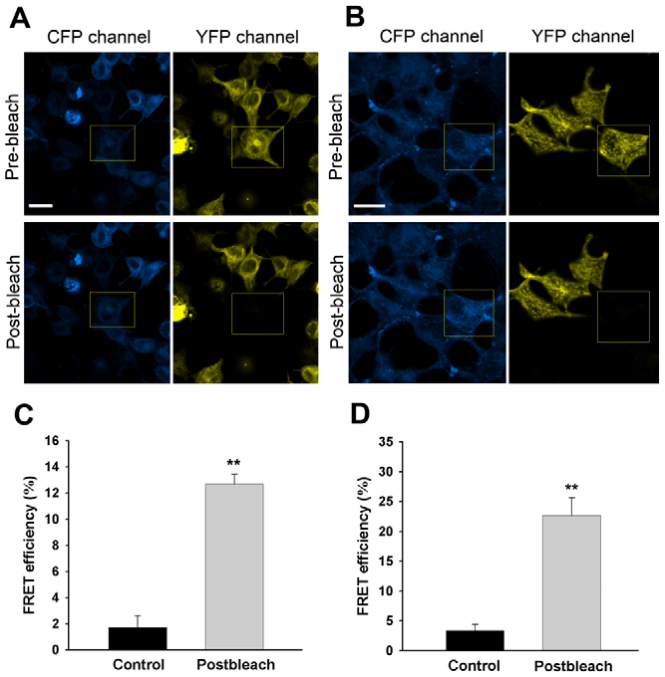
FRET measured by donor dequenching after acceptor photobleaching in HEK293T cells cotransfected with eCFP-STIM1 and eYFP-STIM1. Part A: CFP-STIM1 images before and after photobleaching of the acceptor within the indicated region (Left column); acceptor YFP-STIM1 intensities before and after photobleaching in the indicated region (Right column). Part B: images acquired near the cell adhesion surface after stimulation of cells with 2 µM TG. Part C: the bar graphs representing FRET efficiency (E) are from 20 independent experiments such as those in part A. The efficiency was determined by the acceptor photobleaching method and was measured only in the (acceptor) bleached area. Cells outside the bleached region were used as controls. Part D: the bar graphs representing FRET efficiency (E) are from the 20 independent experiments in part B. All data are represented as mean±S.D. The significance levels indicated are as follows: **P<0.001. Scale bars, 20 µm.

In the current study, FRET measured by donor dequenching after acceptor photobleaching was also utilised to detect the interaction between STIM1s. HEK293T cells were cotransfected with eCFP-STIM1 and eYFP-STIM1 plasmids. After culture, laser scanning microscopy images were taken before and after photobleaching of the acceptor for 15 s with a 514 nm laser beam. Yellow fluorescence quenching and increase in the intensity of blue fluorescence were observed after the photobleaching. The transfer efficiency was calculated using the equation: E = 1–F_pre_/F_post_. As shown in Fig. 3A and C, the average energy transfer efficiency for the cells withphotobleached acceptors in the resting state was 12.7% compared with only 1.7% in the controls. Following TG-induced Ca^2+^ store depletion, the energy transfer efficiency was 22.6% (Fig. 3 B, D).

### Orai1 recruitment to ER-PM junctions depends on STIM1

When expressed by itself in HEK293T cells, mCherry-tagged STIM1 (mCh-STIM1) formed distinct puncta after depletion of Ca^2+^ stores with TG (Fig. S1A). In contrast, Orai1-eYFP expressed alone did not form puncta in response to TG (Fig. S1B). However, coexpression of mCh-STIM1 with Orai1-eYFP restored its ability to form puncta (Fig. S1C). These data suggest that STIM1 recruitment to ER-PM junctions is independent of Orai1, whereas Orai1 recruitment to these sites depends on binding to STIM1 or a STIM1-associated protein, as has been previously suggested [20].

### Orai1 clusters and colocalises with BiFC-STIM1 puncta upon Ca^2+^ store depletion

**Figure 4:**
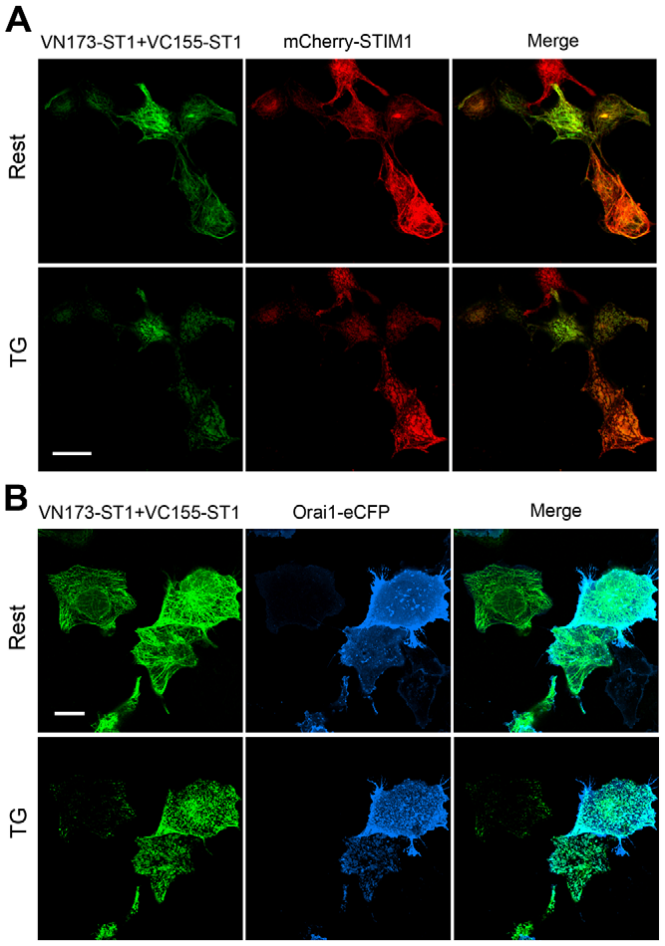
Ca^2+^ store depletion causes relocalisation of coexpressed BiFC-STIM1 and mCherry-STIM1, or coexpressed BiFC-STIM1 and Orai1-eCFP to ER-PM junctions to form colocalised puncta. Part A: HEK293T cells were cotransfected with VN173-ST1, VC155-ST1 and mCh-STIM1 constructs. Live cells were examined under a confocal microscope at the cell footprint. Confocal images of the same cells were taken before (Rest) and 3 min after addition of TG (TG). In resting cells, mCh-STIM1 and BiFC-STIM1 exhibited a colocalised fibrillar distribution (Rest). Ca^2+^ store depletion caused BiFC-STIM1 and mCh-STIM1 to form colocalised puncta. Part B: HEK293T cells were cotransfected with VN173-ST1, VC155-ST1 and Orai1-eCFP constructs. After Ca^2+^ store depletion, Orai1-eYFP accumulated with BiFC-STIM1 as puncta localised near the plasma membrane. Scale bars, 20 µm.

As shown in Fig. 4A, coexpressing VN173-ST1, VC155-ST1 and mCh-STIM1 in HEK293T cells led to an identical ER distribution for all three proteins, as observed in all cellular focal planes using confocal microscopy (Rest). mCh-STIM1 and BiFC-STIM1 (combination of VN173-ST1 and VC155-ST1) appeared to colocalise and exhibited a fibrillar distribution under resting conditions (Rest). After Ca^2+^ store depletion, BiFC-STIM1 and mCh-STIM1 colocalised as puncta near the plasma membrane of HEK293T cells (TG). In order to observe the interaction between Orai1 and BiFC-STIM1, eCFP was fused to the C-terminus of Orai1 (Orai1-eCFP), and this construct was coexpressed with VN173-ST1 and VC155-ST1. Consistent with the previous experiments, TG-induced depletion of Ca^2+^ stores caused punctate redistribution of BiFC-STIM1 near the plasma membrane (Fig. 4B). Following depletion of Ca^2+^ stores, Orai1-eCFP also assembled into large puncta that colocalised with BiFC-STIM1 puncta, as observed by dual-colour confocal microscopy at the cell footprint (Fig. 4B, TG).

### 2-APB prevents or reverses relocalisation of STIM1 oligomers near plasma membrane puncta

**Figure 5:**
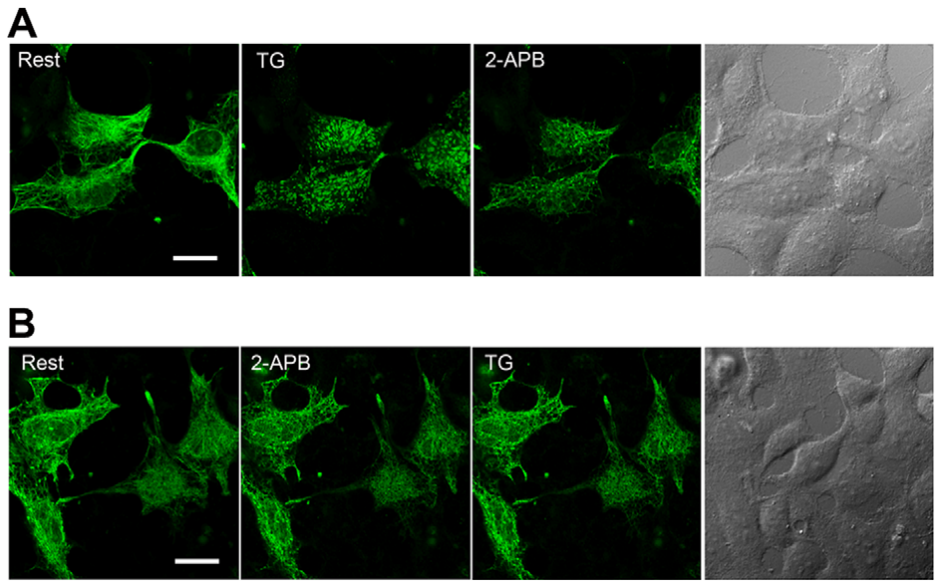
2-APB reverses or prevents BiFC-STIM1 relocalisation to puncta near the plasma membrane. HEK293T cells were cotransfected with VN173-ST1 and VC155-ST1 constructs. After 24 h, live cells were examined under a confocal microscope at the cell footprint. Part A: VN173-ST1 and VC155-ST1 coexpressed in HEK293T cells redistributed from a fibrillar appearance (Rest) to puncta at the cell periphery after Ca^2+^ store depletion with TG. Puncta were visible at the cell footprint. The addition of 50 µM 2-APB rapidly reversed the relocalisation of BiFC-STIM1 to plasma membrane puncta (2-APB). Part B: 2-APB prevented BiFC-STIM1 relocalisation to puncta near the plasma membrane. Confocal imaging was carried out in experiments in which cells expressing BiFC-STIM1 were pretreated with 50 µM 2-APB for 5 min prior to store depletion with TG. Scale bars, 20 µm.

2-APB is a reliable blocker of store-operated Ca^2+^ entry (SOCE). Recent research and our data suggest that the mechanism of inhibition of SOCE by 2-APB can be attributed to its effect on the relocalisation of STIM1 and Orai1. Like wild-type GFP-STIM1, VN173–STIM1 and VC155–STIM1 coexpressed in HEK293T cells exhibited filamentous distribution that localised predominantly to the ER (Fig. 5A, Rest). After cells were exposed to TG, STIM1 oligomers (BiFC-STIM1) took on a punctate appearance (Fig. 5A, TG). After the addition of 50 µM 2-APB, the puncta dispersed completely (Fig. 5A, 2-APB). Furthermore, as shown in Fig. 5B, pretreatment of cells with 50 µM 2-APB largely prevented BiFC-STIM1 from migrating to nearby plasma membrane regions in response to store depletion. These results suggest that the pharmacological properties of BiFC-STIM1 are consistent with those of wild-type GFP-STIM1.

### Validation of the functional properties of BiFC-STIM1 proteins

**Figure 6:**
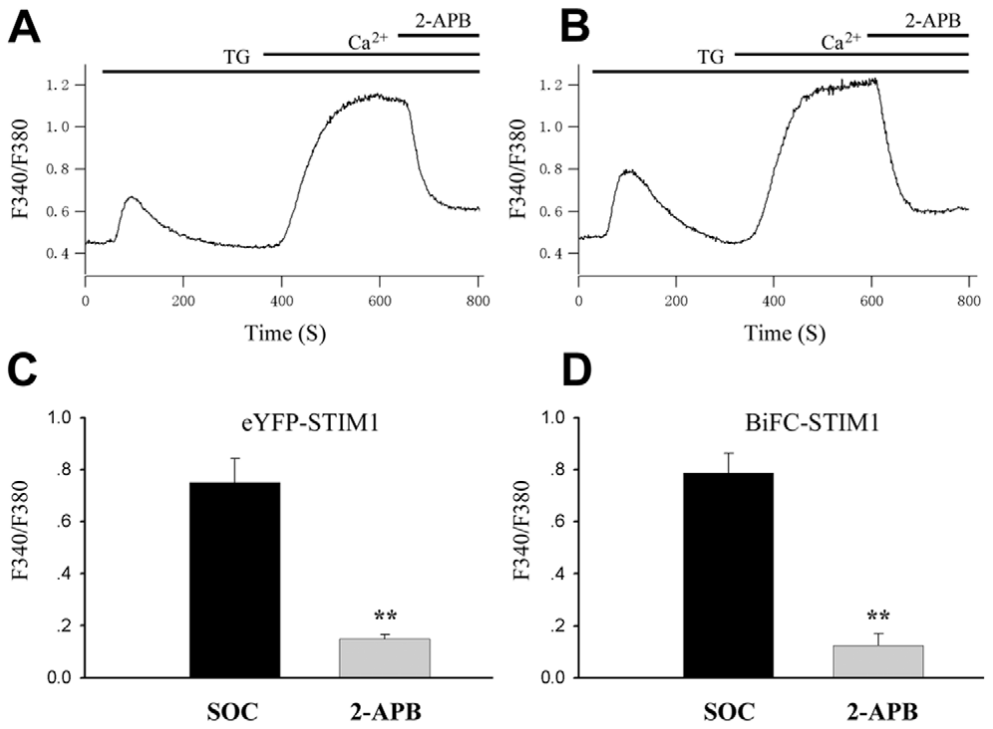
Functional validation of the fusion protein of BiFC-STIM1 in HEK293T cells. SOCE was assayed using Ca^2+^ microfluorimetry in HEK293T cells transfected with eYFP-STIM1 alone or with both VN173-ST1 and VC155-ST1 plasmids. The cells were first treated with TG (2 µM) in a Ca^2+^-free solution to empty the Ca^2+^ stores and subsequently switched to normal extracellular solution containing 2 mM Ca^2+^; this induced a transient increase in the cytosolic Ca^2+^ concentration. Part A: representative trace of Ca^2+^ dynamics in control HEK293T cells overexpressing pH-STIM1. Part B: representative trace of Ca^2+^ dynamics in cells cotransfected with both VN173-ST1 and VC155-ST1. Part C: mean data showing the effect of 50 µM 2-APB on SOCE from experiments such as those shown in A (n = 18 cells; **p<0.001). Part D: mean data showing the effect of 50 µM 2-APB on SOCE in experiments in part B (n = 18 cells; **p<0.001).

In order to verify the function of BiFC-STIM1 protein, we used Ca^2+^ photometry to measure Ca^2+^ entry evoked by store depletion in HEK293T cells expressing the fusion proteins. SOCE in HEK293T cells was analysed using a standard Ca^2+^ add-back assay, whereby intracellular Ca^2+^ stores were depleted by treating cells with the SERCA pump inhibitor, TG in Ca^2+^-free conditions, followed by restoration of extracellular Ca^2+^ to 2mM. Fig. 6A shows recordings of SOCE from HEK293T cells expressing eYFP-STIM1. The addition of 2-APB (50 µM) rapidly blocked the SOCE. As shown in Fig. 6B, 50 µM 2-APB also rapidly blocked SOCE in HEK293T cells cotransfected with both VN173-ST1 and VC155-ST1. As shown in part C and D of Fig. 6, the maximum [Ca^2+^]_i_ elevation (0.75±0.09) evoked by SOCE in HEK293T cells expressing BiFC-STIM1 was same as the maximum [Ca^2+^]_i_ elevation (0.79±0.07) in cells expressing eYFP-STIM1 (n = 18, p>0.05).

## Discussion

Although research in the past 20 years has provided a detailed electrophysiological and pharmacological characterisation of CRAC channels, the precise molecular mechanism by which ER Ca^2+^ depletion activates the CRAC channel remains elusive [5], [21]. Recently, a genome-wide screen using RNA interference led to the identification of STIM1 and ORAI1 as two key molecular components of the I_CRAC_-signalling pathway. It is now clear that STIM1 is a Ca^2+^ sensor located in the ER membrane [22]–[24], whereas ORAI proteins (including Orai1, –2, and –3) are suggested to form highly Ca^2+^ selective channels in the plasma membrane [25]–[27]. Upon Ca^2+^ store depletion, STIM1 redistributes to form puncta close to the plasma membrane and activates ORAI1 channels that manifest the biophysical fingerprint of endogenous CRAC channels [28], [29].

In previous studies, a sequence containing only the EF-hand and SAM domain of STIM1 was recombinantly expressed in *E. coli*, and the effects of Ca^2+^ binding on its conformation were studied in vitro [15]. These biophysical analyses showed that in the presence of Ca^2+^, the EF-hand/SAM domain exists as a monomer that has a compact alpha-helical structure. In contrast, when Ca^2+^ is depleted, the EF hand/SAM domain changes to a less compact conformation that promotes aggregation into dimers and oligomers. Repletion of Ca^2+^ caused these aggregates to revert back to monomers, demonstrating the reversibility of this Ca^2+^-dependent conformational change. However, other reports indicate that STIM1–STIM1 and STIM1–STIM2 interactions do occur in Ca^2+^ store-replete cells because both endogenous and overexpressed STIM1–STIM1 and STIM1–STIM2 complexes can be readily co-immunoprecipitated from store-replete cell lysates [10], [18], [19], [30]. These results were obtained from in vitro experiments, and it is important to note that the conditions in in vitro experiments are not identical to those in living cells.

We utilised the technique of bimolecular fluorescence complementation (BiFC) to identify and visualise the state of STIM1 molecules in living cells in the resting state. If VN173-ST1 interacts with VC155-ST1, the interaction would bring VN173 and VC155 into close proximity and an intact Venus molecule would be reconstituted allowing direct visualisation of the oligomeric state of STIM1. In our experiments, coexpression of VN173-ST1 and VC155-ST1 led to strong fluorescence emission, suggesting the presence of STIM1 oligomer (BiFC-STIM1), possibly as a dimer. We found that in resting cells, BiFC-STIM1 exhibited a fibrillar distribution similar to the distribution of wild-type GFP-STIM1. BiFC-STIM1 did not constitutively form puncta beneath the plasma membrane, which suggests that higher order oligomers rather than lower order oligomers (such as dimers) of STIM1 have the ability to migrate to ER-PM junctions to form aggregates. Ca^2+^ store depletion induced BiFC-STIM1 to relocate to ER-PM junctions and form puncta. 2-APB, a reliable blocker of SOCE, prevented and reversed the relocalisation of BiFC-STIM1 to puncta near the plasma membrane. Our results also demonstrate that both the functional and pharmacological properties of BiFC-STIM1 were not altered compared with those of wild-type STIM1. These data indicate that BiFC-STIM1 is identical to wild-type STIM1 in morphological characteristics, dynamics, drug properties and functions. In addition, to further examine the state of STIM1 in resting cells, we used acceptor-photobleaching-based FRET to analyse the interactions of STIM1 in resting cells. HEK 293T cells coexpressing CFP/YFP-tagged STIM1 exhibited comparably higher FRET efficiency in the resting state, which suggests that the interaction between STIM1s exist in resting cells. Thus, using direct visualisation of STIM1 molecule interactions, we demonstrate for the first time that STIM1 exists in an oligomeric form in living cells at rest. Although the functional significance of the oligomeric form of STIM1 in resting cells remains unclear and needs further investigation, we propose that the oligomeric structure of STIM1 in the resting state is required for its rapid transition from lower order oligomers (such as dimers) to higher order oligomers upon Ca^2+^ store depletion. In addition, the oligomeric state of STIM1 in resting cells may be important for its rapid constitutive movement along microtubules [18].

STIM1 contains two Ca^2+^-binding EF-hand motifs with a single sterile alpha-motif (SAM) domain on the luminal side of the ER. The EF hand motif of STIM1 is, as expected, responsible for Ca^2+^ sensing in the ER lumen [15]. Deletion of the SAM domain alone renders STIM1 incapable of Ca^2+^ store depletion-mediated puncta formation and does not result in constitutive STIM1 aggregation as puncta nor constitutive activation of the SOC channel [18]. These data suggest that the SAM domains of STIM1 play a critical role in determining the Ca^2+^-dependent conformational transition of the luminal region. Beyond the single conserved transmembrane (TM) segment, STIM1 has two coiled-coil regions that overlap with an ezrin-radixin-moesin (ERM)-like domain, followed by a divergent region that contains multiple serine and proline residues (S/P rich) and lysine residues (K rich) in the cytoplasmic side. Previous studies indicate that the cytoplasmic domain mediates STIM1 aggregation and SOC activation. Three recent studies have independently identified a crucial Orai-recruiting and Orai-activating domain within the ERM domain of STIM1; it was named the CRAC-activating domain CAD (CAD) [29], STIM–Orai-activating region (SOAR) [31], and Orai-activating small fragment (OASF) [32]. In our experiments, mutant STIM1 was used to detect the site of protein-protein interactions in living cells. We found that cells coexpressing VN173-ΔSAM and VC155-ΔSAM (SAM domains deletion mutants) still exhibited high BiFC efficiency. However, cells cotransfected with VN173-ΔC and VC155-ΔC (cytoplasmic coiled-coil/ERM domain deletion mutants) did not exhibit yellow fluorescence, suggesting that the cytoplasmic coiled-coil/ERM domain is necessary for STIM1 oligomerisation in cells in the resting state as has been suggested [17], [18], [30]. Our findings are different from those of Stathopulos, who reported that STIM1 exists only in a monomeric form in the resting state in vitro [15]. The discrepancy between our results and theirs may be due to the different-length of STIM1 protein fragments studied. In Stathopulos’ experiment, the STIM1 protein fragments studied contained only the EF-hand pairs and SAM domain; the C-terminal of STIM1 (coiled-coil/ERM domain) was not included. In contrast, in our experiment, full-length STIM1 was used to detect protein-protein interactions in living cells. Above all, our data provide important advances in the understanding of subunit stoichiometry of CRAC channels and the mechanisms of transmission of information from STIM1 to Orai1.

## Supporting Information

Figure S1 **Orai1 recruitment to puncta at the ER-PM junctions depends on binding to STIM1**. Part A: mCh-STIM1 expressed alone in HEK293 cells redistributes from a diffuse ER distribution (Rest) to the cell periphery after Ca^2+^ store depletion with TG. After TG treatment, puncta are visible at the cell footprint. Part B: Orai1-eYFP expressed alone does not redistribute into puncta after Ca^2+^ store depletion. Part C: when expressed together, mCh-STIM1 and Orai1-eYFP form colocalised puncta after Ca^2+^ store depletion. Scale bars, 20 µm.
(TIF)
