# Supplementary figures and images for: Visualisation and Identification of the Interaction between STIM1s in Resting Cells
Source: PLoS One. 2012 Mar 16;7(3):e33377. doi: 10.1371/journal.pone.0033377 (PMC3306384; doi:10.1371/journal.pone.0033377)

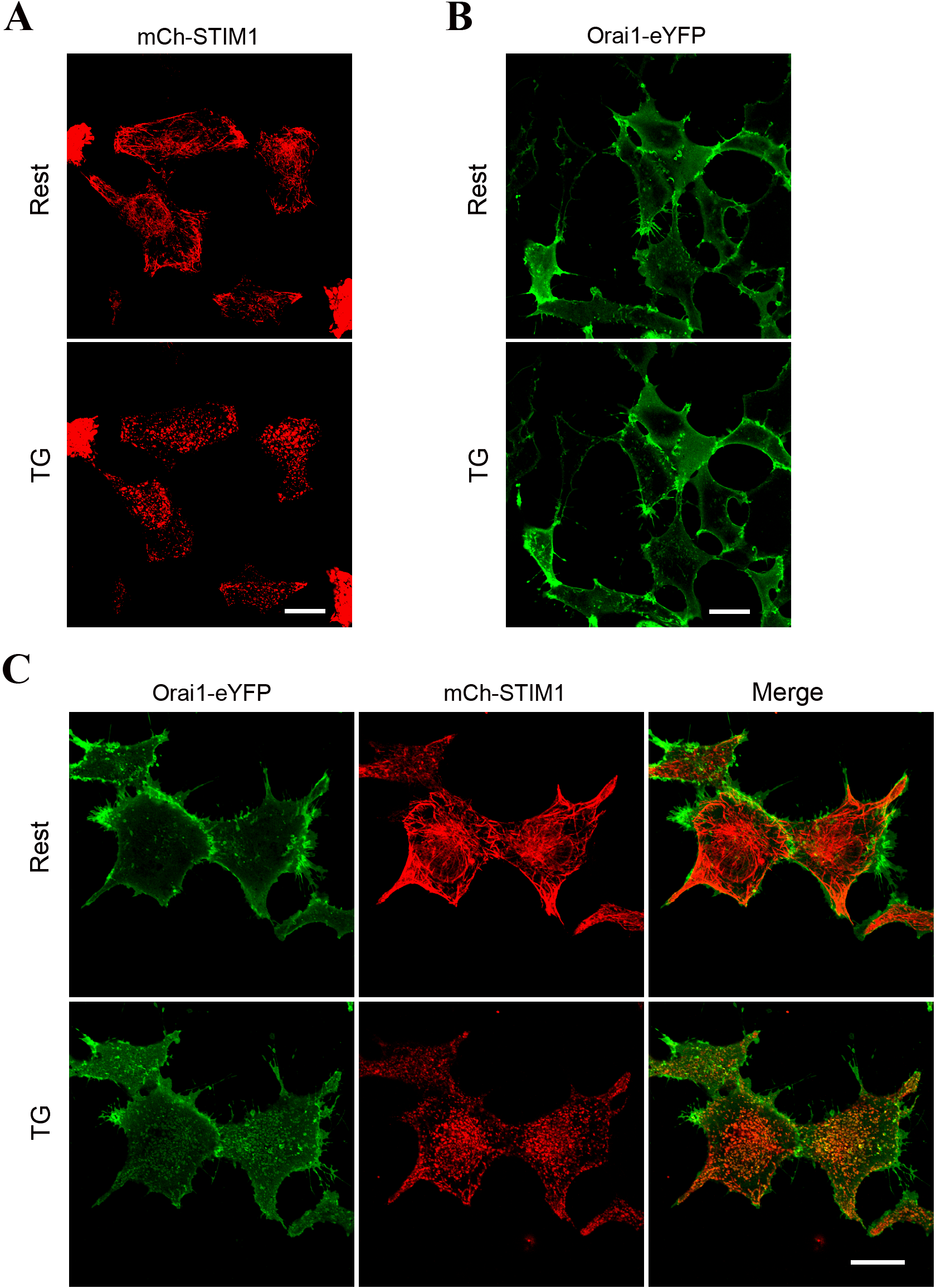

Supplement: Figure S1 — Orai1 recruitment to puncta at the ER-PM junctions depends on binding to STIM1. Part A: mCh-STIM1 expressed alone in HEK293 cells redistributes from a diffuse ER distribution (Rest) to the cell periphery after Ca2+ store depletion with TG. After TG treatment, puncta are visible at the cell footprint. Part B: Orai1-eYFP expressed alone does not redistribute into puncta after Ca2+ store depletion. Part C: when expressed together, mCh-STIM1 and Orai1-eYFP form colocalised puncta after Ca2+ store depletion. Scale bars, 20 µm. (TIF) [file pone.0033377.s001.tif]
